# Kinetics of Antibody Binding to Membranes of Living Bacteria Measured by a Photonic Crystal-Based Biosensor

**DOI:** 10.3390/bios6040052

**Published:** 2016-10-11

**Authors:** Ekaterina Rostova, Carine Ben Adiba, Giovanni Dietler, Sergey K. Sekatskii

**Affiliations:** Laboratoire de Physique de la Matière Vivante, IPHYS, École Polytechnique Fédérale de Lausanne (EPFL); Rte de la Sorge, 1015 Lausanne, Switzerland; carine.benadiba@epfl.ch (C.B.A.); giovanni.dietler@epfl.ch (G.D.); serguei.sekatski@epfl.ch (S.K.S.)

**Keywords:** photonic crystal, label-free biosensor, bacteria, binding kinetics, antibody, ligand-receptor interaction

## Abstract

Optical biosensors based on photonic crystal surface waves (PC SWs) offer a possibility to study binding interactions with living cells, overcoming the limitation of rather small evanescent field penetration depth into a sample medium that is characteristic for typical optical biosensors. Besides this, simultaneous excitation of *s*- and *p*-polarized surface waves with different penetration depths is realized here, permitting unambiguous separation of surface and volume contributions to the measured signal. PC-based biosensors do not require a bulk signal correction, compared to widely used surface plasmon resonance-based devices. We developed a chitosan-based protocol of PC chip functionalization for bacterial attachment and performed experiments on antibody binding to living bacteria measured in real time by the PCSW-based biosensor. Data analysis reveals specific binding and gives the value of the dissociation constant for monoclonal antibodies (IgG2b) against bacterial lipopolysaccharides equal to *K_D_* = 6.2 ± 3.4 nM. To our knowledge, this is a first demonstration of antibody-binding kinetics to living bacteria by a label-free optical biosensor.

## 1. Introduction 

According to reviews on antimicrobial resistance, each year 700,000 people die of infections caused by drug-resistant bacteria. A continuous spread of resistance may lead to 10,000,000 deaths per year by 2050, which will exceed cancer-related deaths [1]. Unfortunately, a substantial amount of antibiotics is misused, and almost 20% of prescribed drugs lack a known mechanism of action [2]. Key drug properties can be understood from kinetics measurements of its binding to a target under conditions which are as close as possible to a native cell environment. Correspondingly, there is a need for an efficient method that enables fast time-resolved measurement of binding interactions*.* Label-free optical biosensors based on various transducers are actively entering this field [3,4]. In recent years, attempts were made to measure binding kinetics on living bacteria by optical surface plasmon resonance (SPR)-based biosensors [5]. Nevertheless, the experiments were not fully successful because of an insufficient penetration depth of the sensing evanescent field into living bacteria. Waveguide sensors based on long-range surface plasmons (LRSP) have a potential to detect binding events on the bacterial surface [6], however, it was not yet demonstrated experimentally. 

Biosensors based on metal-free photonic crystals (PCs) support electromagnetic surface waves (SWs) with a deeper penetration depth into the sample medium [7] offering a solution for the sensing of the binding interactions with living bacterial and, possibly, also eukaryotic cells. Besides a deeper penetration depth into the liquid sample, the PC-based biosensors have the main advantage of supporting at the same wavelength of two surface waves that differ much in their penetration depth (impossible in SPR-based sensors). This sensor feature permits unambiguous separation of surface and volume contributions to the measured signal. Thus, PC-based biosensors do not require a bulk signal correction caused by a refractive index variation compared to widely used SPR-based devices. Moreover, the PC-based biosensors offer several other benefits such as tailoring of the PC to a desired laser wavelength, excitation of several surface modes at the same wavelength, lower losses in the dielectric material and, therefore, higher sensitivity, reusability of the PC chip, and flexible glass surface chemistry. 

**Figure 1:**
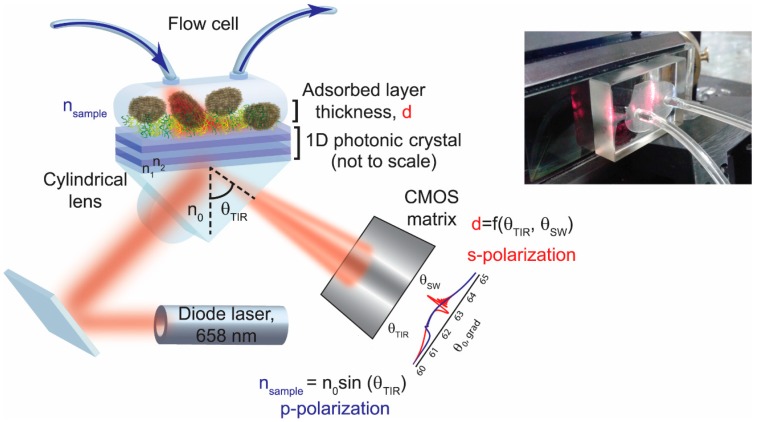
A sketch of the label-free biosensor used for simultaneous measurements of surface and volume effects and based on angular interrogation of two photonic crystal surface waves (PC SWs). The biosensor employs a specially designed PC (layers of SiO_2_ with a refractive index n_1_ alternating with Ta_2_O_5_ layers with a refractive index n_2_) supporting the surface waves with a different penetration depth into the sample medium. The surface waves are excited at 658 nm using a standard Kretschmann configuration. The sensor response is obtained by identifying and tracking the resonance dip of these two surface waves. The inset: a photograph of the flow cell and the photonic crystal behind it attached to the prism via a refractive index matching oil. See explanation of the formulas in the text.

Recently, an implementation of a specially designed PC supporting long-range propagation surface waves permitted the increase of the mass device sensitivity to the level of 0.3 pg/mm^2^ [8]. The PC is a periodic structure of alternating dielectric layers having different refractive indices (RIs) which create a periodic modulation of the RI on the light wavelength scale. The PC-supported electromagnetic surface waves are excited at a certain wavelength in the photonic band gap caused by destructive interference of the incident light due to multiple reflections from the boundaries, and propagate along the PC—external medium interface being strongly confined there. In the biosensor, such an interface is formed between a functionalized photonic crystal chip surface and a dispersed biological sample in a liquid. The binding kinetics are measured by monitoring the changes in the optical properties of electromagnetic surface waves decaying exponentially away from the interface (Figure 1, [8]). Both *s*- and *p*-polarized surface waves with different penetration depths into the sampling medium are supported by this biosensor. These two waves are utilized to measure simultaneously and independently a liquid refractive index *n_sample_* and an adsorbed layer thickness *d* (Figure 1). The former is determined directly from the total internal reflection (TIR) angle for the *p*-polarized surface wave, while the excitation angle of the *s*-polarized surface wave is recalculated to the thickness of the layer adsorbed during the binding experiment. The adsorbed layer thickness is defined as an average of an adsorbed ligand layer over a detectible surface of the PC. The signal is collected from the area of approximately 100 μm × 1.5 mm, implying the averaging over thousands of bacteria. 

This article focuses on application of the optical biosensor employed a PC for excitation of SWs to study binding kinetics of antibodies to a bacterial surface. We describe an experimental protocol for the binding experiment, data handling and a binding model, and then discuss the experimental data on antibody binding kinetics and further data analysis including calculation of the dissociation constant. The performed experiments successfully demonstrate application of the PC biosensor to study binding kinetics of antibodies to living bacteria. 

## 2. Materials and Methods 

### 2.1. Biosensor Setup (Figure 1)

The key component of the PC SW biosensor is a PC chip consisting of three layers of SiO_2_ with a thickness of 183.2 nm (with a RI n_1_ = 1.47) alternating with three Ta_2_O_5_ layers of 111.2 nm (with a RI n_2_ = 2.1) terminated with a SiO_2_ of 341.6 nm (Figure 1). This seven-layer structure is deposited onto *BK-7* glass slide by magnetron sputtering. This chip is specially designed to support *s*- and *p*-polarized surface waves excited at total internal reflection conditions in Kretschmann configuration. These two surface waves are excited at the interface between the photonic crystal and the sample medium by a diode laser at the wavelength of 658 nm. The laser beam cross-section is expanded to 1 cm by a cylindrical lens to cover a large area of the biosensor chip surface. The focused laser beam entering a glass prism is reflected under the total internal reflection condition and detected on a CMOS camera in real time every 0.2 s. The excited surface waves propagate along the surface of the biosensor PC chip opposite to the one attached to the prism with a refractive index matching oil. The excitation of the surface waves is manifested as two dips in the intensity profile of the reflected laser radiation detected on the camera matrix: *p*-polarized wave excited at TIR angle at 61.54° (Figure 1, blue curve) and *s*-polarized wave excited at the resonance angle of 62.63° (Figure 1, red curve). First, the software tracks changes of the critical angle and resonance dip positions in the intensity profile along the camera pixels, and converts them into the angular changes $\Delta \theta_{TIR}$ and $\Delta \theta_{SW}$. An angle here is defined as an angle between a normal vector to the PC surface and a reflected beam direction where a surface wave is observed. Next, the angle change $\Delta \theta_{TIR}$ is recalculated to the change of the bulk refractive index of the liquid sample $n_{sample}$ directly from the TIR angle $\theta_{TIR}$ in this sample as it is done in critical-angle Abbe refractometers [9]: $n_{sample}=n_{0}\sin(\theta_{TIR})$, where *n*_0_ is the prism refractive index (1.52).

Changes in the adsorbed layer thickness *d* are derived from changes of the resonance angle $\Delta \theta_{SW}$ and ones of the sample refractive index $\Delta \;n_{sample}$, assuming that the RI of a single bacteria is equal to 1.388 [10]. Thus, the adlayer thickness *d* is calculated as a function of the measured angles $\Delta \theta_{TIR}$ and $\Delta \theta_{SW}$: $d=f(\Delta \theta_{TIR},\;\Delta \theta_{SW})$*.* A linear method based on the Taylor expansion [7,11] is used here for the conversion of the dip positions on the intensity profile on the camera matrix to the adlayer thickness. If we introduce an angular parameter for the surface wave *ρ* such as $\Delta \rho =\;n_{sample}\;\Delta \theta_{SW}$ its changes can be expressed as follows: $\Delta \rho =\;\frac{\partial \rho }{\partial n_{sample}}\Delta n_{sample}+\frac{\partial \rho }{\partial d}\Delta d$. From this expression, the adlayer thickness is derived: $\Delta d=\;\frac{\Delta \rho -\partial \rho /\partial \;n_{sample}\;\Delta n_{sample}}{\partial \rho /\partial \text{​ }d}$. The partial derivatives of the angular parameter are calculated using a dispersion relation for one-dimensional photonic crystal [7,12].

The biosensor response is measured while a bacterial suspension is pumped through the flow cell (Figure 1) of a 100 µL volume. When the bacteria are captured by the functionalized chip surface, the local refractive index at the chip surface increases and, therefore, the propagation constants of the surface waves change leading to the shift of the resonance dip positions along the camera screen pixels. An analogue of the reported biosensor currently is commercially available as “EVA 2.0” device, see www.pcbiosensors.com.

### 2.2. Experiment Protocol

**Figure 2:**
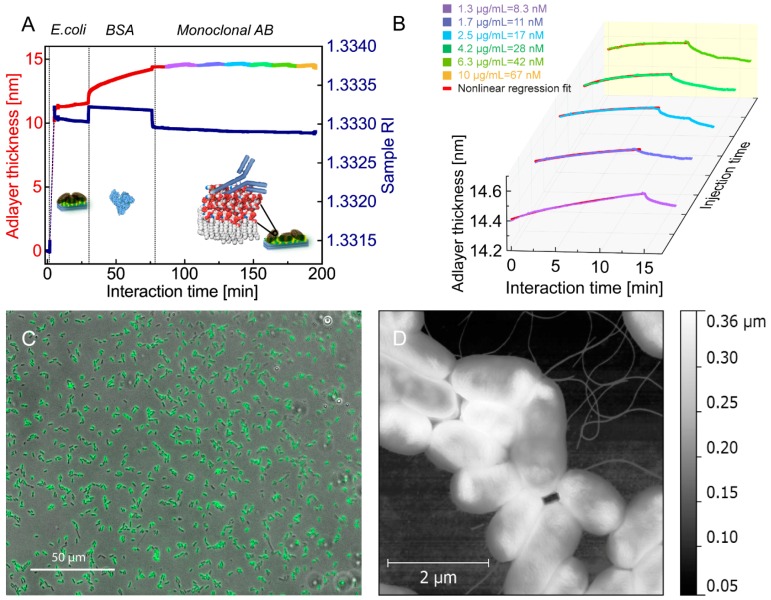
Binding kinetics of monoclonal antibodies against lipopolysaccharide (LPS) to living bacteria *E. coli* measured by a photonic crystal-based biosensor. (**A**) A typical sensorgram of *E. coli* attachment (first five minutes), blocking by bovine serum albumin (BSA) (after 30 min), and binding kinetics of antibodies against lipopolysaccharides to living bacteria (after 87 min, colored curves). Here, a simultaneous measurement of the adsorbed layer thickness (left red axis) and bulk refractive index (right blue axis) takes place, see also Figure 1 for the biosensor setup. The inset cartoons depict BSA and antibody on the bacterial membrane; (**B**) antibody binding measured at different concentrations: association and dissociation phases, 15–20 min between injections of antibody solutions. The association phases are fitted by a nonlinear regression algorithm (see explanation in the text), the fitting curve is displayed red; (**C**) immunofluorescence imaging of unlabeled primary mouse monoclonal antibodies (targeting LPS of *E. coli* during the experiment in vitro) recognized by anti-mouse secondary antibodies Alexa 488 dye-conjugated (attached onto the chip after the experiment), obtained by wide-field epifluorescence microscopy; (**D**) a topography atomic force microscopy (AFM) image of *E. coli* with flagella attached to the photonic crystal chip (performed in air after the binding experiment).

The utilized biosensor technology requires a functionalization coating of high optical quality and high adherent capabilities to bacteria. The chip functionalization protocol was developed to obtain a high density of captured bacteria and, therefore, higher signal-to-noise ratio and cell variety. The chip was first treated by air-plasma and then chitosan coated (adapted from [13]). Next, bacteria *E. coli* *DH5α* dispersed in phosphate-buffered saline (PBS) were attached to the functionalized chip under a continuous flow. The optimal flow rate corresponded to 100 μL/min; this rate enables us to avoid mass-transport limitation but to keep the flow laminar [14]. After approximately 5 min, the flow cell was rinsed with the pure buffer to remove loosely bound bacteria. Bacteria-free areas were further blocked with BSA (bovin serum albumin) in PBS solution, and afterwards rinsed with the buffer. Finally, a solution of specific antibodies was circulating in the flow cell for 10 min to favor association of the antibodies to the captured bacteria (Figure 2A,B). The subsequent rinsing with the PBS buffer resulted in dissociation of specifically bound antibodies to avoid a regeneration phase. The last two steps were repeated for different antibody concentrations from the lowest (1.25 μg/mL or 8.3 nM) to the highest one (10 μg/mL or 67 nM) to obtain a concentration series. After the last association phase, the flow cell was rinsed shortly with the buffer and ultrapure water to keep the antibodies bound for the immunostaining. 

After the binding experiment, the chip with attached bacteria and antibodies was air-dried and examined by atomic force microscopy (AFM) (in air) and wide-field epifluorescence microscopy (Figure 2C). The chip with bacteria and unlabeled primary mouse monoclonal antibodies was incubated for 45 min with Alexa 488 dye-conjugated anti-mouse secondary antibodies, and then thoroughly rinsed with PBS. Imaging was performed on an inverted optical microscope Axiovert 200M with a 40× objective (NA 0.65, Carl Zeiss, Oberkochen, Germany) equipped by a CoolSnap HQ2 camera (PhotoMetrics) and the multi-dimensional acquisition module of the software Meramorph (Molecular Devices).

### 2.3. Reagents

Nonpathogenic bacteria *E. coli* serotype *DH5α* strain *K-12* were grown in the culture medium Luria broth (LB) for 16 h to arrest the cells in the exponential phase. To remove the medium, the suspension was centrifuged 3 times at 3000 rpm (Hitachi CT15RE High-Speed Micro Centrifuge) and dispersed in PBS (phosphate-buffered saline) 1X to the final concentration of 10^9^ cell/mL. Bovin serum albumin (BSA) was purchased from Sigma, dispersed in PBS 1X to the concentration of 1 mg/mL for the experiment with monoclonal antibodies and 10 mg/mL for polyclonal ones. Mouse monoclonal antibodies IgG2b isotype against lipopolysaccharides (GeneTex, Irvine, CA, USA) and polyclonal goat antibodies IgG against lipid A (GeneTex) were dispersed in PBS 1X. Ultrapure water with conductivity of 0.055 µS/cm (Blanc Labo, Lonay, Switzerland) was used for rinsing. For immunostaining, secondary anti-mouse antibodies conjugated with Alexa Fluor 468 were purchased from GeneTex. All reagents were used as received without additional treatment.

### 2.4. Data Handling and Binding Model 

**Figure 3:**
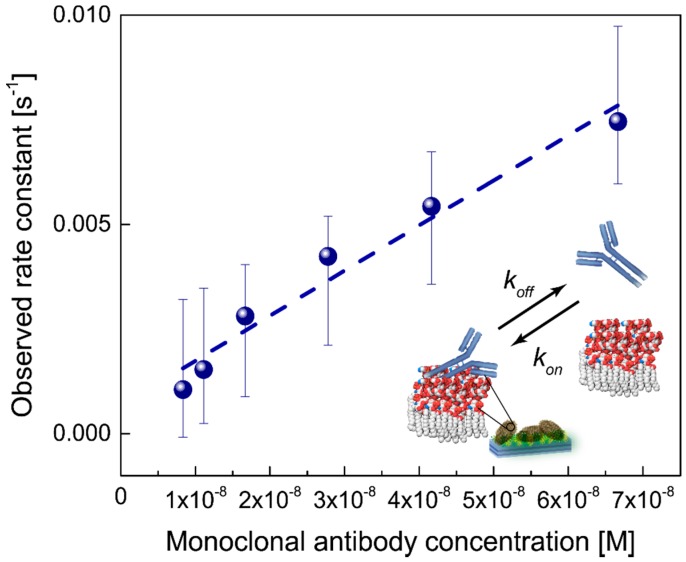
Monoclonal antibody binding. A one-to-one binding model was used to fit the data and obtain the observed rate constant *k_obs_* as a parameter (see explanations in the text). This rate constant was subsequently plotted as a function of the antibody concentration and fitted to the straight line for monoclonal antibodies. From the data fit, the dissociation constant for monoclonal antibodies bound to living *E. coli DH5α* was equal to 6.2 ± 3.4 nM. The confidence limits for the predicted linear fit were calculated using a classical formula for the confidence interval, which has 95% chance to contain the true parameter value.

Association binding phases measured at different antibody concentrations were fitted to one-to-one binding model (first-order binding kinetics). Besides binding reversibility, this model assumes that all binding sites are equal and that each new binding does not alter affinity properties of the ligand or receptor. The association phase (Figure 2B) corresponding to a different antibody concentration was fitted to the model (red curves) using a freely available software package SimFit (“Simfit: simulation, statistical analysis, curve fitting and graph plotting”, [15]) based on an unweighted nonlinear regression algorithm [16]. The binding model was described by a single exponential function with monotonic growth from a baseline: $d=A\;(1-e^{-k_{obs}t})+C$, where *d* is an adsorbed layer thickness (nm); *A* is a constant; *C* is a baseline constant; and $k_{obs}$ is the observed rate constant (s^−1^) [16]. If nonspecific binding takes place, another exponential function should be added. In a one-to-one binding model, the observed rate constant linearly depends on the antibody concentration as $k_{obs}=k_{on}\;C_{antibody}+k_{off}$, where *k_on_* and *k_off_*—association and dissociation rate constants, respectively—and *C_antibody_* is an antibody concentration (measured in moles per liter, M; considered IgG molecular mass of 150 kDa). Thus, in the first-order kinetics model, the observed rate constant as a function of the antibody concentration should follow a linear dependence. The binding strength of an antibody to a single epitope is characterized by the binding affinity *K_A_* or dissociation constant *K_D_ = k_off_/k_on_ = 1/K_A_* (in M). For the *k_obs_* data fitting, a linear regression analysis was used based on the ordinary least squares method. Along with the linear plot parameters, the confidence limits for the predicted linear fit were calculated (Figure 3) using a classical formula for the confidence interval [17], which has 95% chance to contain the true parameter value. A classical measure of the relative fit quality in statistics, Akaike information criterion (AIC) [16,17], was applied to estimate goodness-of-fit, which serves as a strong evidence for a correctly chosen statistical model.

## 3. Results and Discussion 

### 3.1. Binding of Bacteria E. coli DH5α

The photonic crystal (PC)-based biosensor was used to study binding kinetics of antibodies to living bacterial cells *E. coli DH5α*. *E. coli* *DH5a* *K-12* strain belongs to Gram-negative bacteria, which have a cell wall formed by a peptidoglycan layer covered by the membrane composed of lipopolysaccharides (LPS). Core oligosaccharides of LPS are negatively charged due to the presence of phosphate residues and sugar acids like ketodeoxyoctonate (KDO). A positively charged chitosan coating of the PC favors bacterial attachment by electrostatic interactions. The experiment shows fast and nonreversible attachment of the bacterial cells (5 min duration, Figure 2A), thus supporting the electrostatic nature of the attachment. Imaging of the chip by AFM (Park Systems, non-contact mode was used) after the experiment shows homogenously attached bacteria (Figure 2D). The AFM images processed by a free software *Gwyddion* give an average bacterial height of 100–150 nm. The average adlayer thickness measured by the biosensor attained 12 nm, which is not equal to the average bacterial cell height (Figure 2D), since the chip surface is not fully covered by the bacteria. 

### 3.2. Mono- and Polyclonal Antibody Binding

Antibodies are soluble proteins produced by B lymphocytes and plasma cells in response to a pathogen. The binding of specific antibodies to an antigen (such as bacterial LPS) is mainly determined by hydrogen-bonds and van der Waals interactions. Specific antibody binding to the recognition site of LPS is a reversible process, and binding strength is characterized by the dissociation constant *K_D_*, measured here by the optical biosensor. 

While the bacteria are attaching to the chip surface, the bulk refractive index of the bacterial suspension sharply increases as the adlayer thickness does. After *5* min of bacterial attachment, the flow cell was rinsed with a PBS buffer for 25 min to remove loosely attached bacteria. During the experiment one may notice that the RI is slowly decreasing, which can be caused by metabolic processes in living bacteria. The adlayer thickness is not biased by such RI changes that are not associated with the ligand attachment because the surface and bulk refractive indices are separately measured in the biosensor due to excitation of two surface waves (see above). After 30 min of the experiment, a solution of BSA in PBS buffer (1 mg/mL) of a higher RI was injected and was passing though the flow cell until the 76th min of the experiment (association of BSA). At this experimental step, BSA macromolecules were attaching onto the chip surfaces that are free of bacteria followed by a PBS rinsing (dissociation of BSA). After 87 min of the experiment, a solution of the lowest monoclonal antibody concentration (1.3 µg/mL) with a lower RI is injected and followed by a concentration series of the antibody. It is worth noting that the average adsorbed layer of bacteria is hundred times thicker than the average layer of antibodies formed onto bacteria surfaces, which again confirms tight attachment of bacteria and attests sufficient sensor sensitivity (Figure 2). The binding curves of the monoclonal antibodies with a different concentration obtained after the 87th min of the experiment are plotted over interaction time (Figure 2B, displayed by different colors) with the same temporal origin in a 3D graph. The *z*-axis “Injection time” is served only to distribute the binding curves on the plot. The yellow curve corresponding to the antibody concentration of 10 µg/mL on Figure 2A is not presented on Figure 2B because the last dissociation phase was omitted to preserve the bound antibodies for the subsequent immunostaining. The immunostaining confirmed binding specificity of the primary antibodies to the bacteria recognized by the fluorescent secondary antibodies (Figure 2C).

After the attachment of bacteria onto the biosensor chip, the association phase of the antibody binding curves measured at different concentrations were fitted using a nonlinear regression method (see the Section 2). The *R*^2^ square of the correlation coefficient between the data and the best fit ranged from 0.98 to 0.99, quantifying the goodness of the fit. 

Though antibodies are produced to be specific to a bacterial membrane, nonspecific binding of the antibodies is indeed always present. Compared to specific binding, nonspecific binding does not reach an equilibrium over time as specific binding does. In this case, total binding will consist of specific and nonspecific binding with observed rate constants *k_obs_* of different orders of magnitude. Therefore, the corresponding binding curves cannot be fitted by a single exponential, but rather by sum of two exponential functions with a different exponent *k_obs_*. In our experiments, we first decreased nonspecific binding of antibodies to the functionalized surface by blocking it with bovine serum albumin (BSA). Next, we verified that the binding curves (Figure 2B) can be perfectly fitted by a single exponential function. After the association phase, almost all bound antibodies dissociated thus confirming binding reversibly and specificity. Finally, the biosensor chips after the experiments were incubated with secondary fluorescent antibodies to verify that primary antibodies were attached selectively to the bacteria during the experiment (Figure 2C).

The rate constants were plotted as a function of the antibody concentration and fitted further to a straight line (Figure 3). The dissociation constant *K_D_* was calculated as a ratio of the line intersect with *y*-axis to the line slope. The dissociation constant for monoclonal antibodies bound to living *E. coli DH5α*, as calculated from the data fit, was equal to 6.2 ± 3.4 nM. This value is of the same order of magnitude as the corresponding values characteristic for many other monoclonal antibodies—lipid antigen interactions [18]. It is also in line with the earlier studies performed on *single* bacteria *E. coli* O157:H7 and polyclonal antibodies Ab157, where the dissociation constants in the range of 10^−10^–10^−8^ M were reported ([19]; in our opinion, due to the small surface plasmon–polariton penetration depth into an external media, the values obtained in those experiments were in all probability measured on the cell edges). 

We also performed similar experiments on binding of *polyclonal* antibodies against lipid A, the toxic moiety of LPS. Unlike monoclonal antibodies, polyclonal ones consist of antibodies that recognize different epitopes of an antigen and therefore have different binding affinities [20]. Experimental results (see the Supplementary Materials, Figure S3) show that these antibodies almost certainly have rather close values of the association rate constant *k_on_* as was attested by the possibility to fit the binding curves at different concentrations by a single exponential function. However, though this simple model well described each individual binding curve (i.e., that pertinent to one certain concentration), the observed rate constant *k_obs_* does *not* obey a linear dependence on the polyclonal antibody concentration. Consequently, we were unable to determine a single binding constant for the entire population of polyclonal antibodies from the kinetic data. Indeed, this is not surprising because, again, each subpopulation of the antibodies here has a different binding constant. The earlier reported binding constants *K_D_* of polyclonal IgG antibodies to fixed bacteria *E. coli O157* are ranged from 10^−7^ M to 10^−6^ M (as measured by a BIAcore [5]). 

## 4. Conclusions 

In this work we presented the photonic crystal (PC)-based biosensor as a versatile, simple, and rapid tool to study binding kinetics of antibodies to *living bacterial cells*. In particular, we have measured the kinetics of the binding of monoclonal antibodies at concentrations ranging from 1.25 μg/mL to 10 μg/mL. The dissociation constant for monoclonal antibodies bound to living *E. coli DH5α* equal to 6.2 ± 3.4 nM has been obtained, and this value is consisted with other studies. 

It is also instructive to compare our experimental data with the data obtained on *isolated bacterial membranes*. For the latter, the binding constants *K_D_* of monoclonal antibodies WN1 222-5 to isolated lipopolysaccharides membranes *E. coli* R-core types ranging from 10^−8^ to 10^−5^ M were reported (as measured by BIAcore and isothermal titration microcalorimetry [21]), which is essentially larger than measured by us for the whole intact living cells. We believe that this discrepancy may be caused primarily by different accessibility of the binding sites within the pool of membrane LPS molecules for possible intermolecular interactions, and that this circumstance should be taken into account when analyzing the results of studies on isolated bacterial membranes. 

In our opinion, the approach reported here to obtain quantitative kinetic information, collected in real time on living cells, can be used not only for the fundamental studies of binding interactions. Apparently, it might also accelerate and optimize screening of drug candidates and even serve as an indispensable tool in antibody production, enabling rapid selection of the best isoforms based on their kinetic properties. Not only antibodies, but various antimicrobial drugs as well, are potential ligands to be explored. It seems quite promising to examine the applicability of this photonic crystal surface waves-based label-free biosensor also for the study of eukaryotic cells (including, possibly, certain cancer cells) which remains our next focus. Ultimately, it can be employed to study the binding of drugs together with a viability test, eventually leading to the identification of currently unclear mechanisms of drug action. 

## Supplementary Materials

The following are available online at www.mdpi.com/2079-6374/6/4/52/s1, Figure S1: A single exponential binding model, Figure S2: Verification of bacterial viability after the experiment, Figure S3: Heterogeneity of antibody binding constants within an antibody population.
