# Supplementary material for: Kinetics of Antibody Binding to Membranes of Living Bacteria Measured by a Photonic Crystal-Based Biosensor
Source: Biosensors (Basel). 2016 Oct 11;6(4):52. doi: 10.3390/bios6040052 (PMC5192372; doi:10.3390/bios6040052)
Supplement: Supplementary file 1 [file biosensors-06-00052-s001.pdf]

Supplementary Materials: Kinetics of Antibody Binding to Membranes of Living Bacteria Measured by a Photonic Crystal-Based Biosensor

Ekaterina Rostova, Carine Ben Adiba, Giovanni Dietler and Sergey K. Sekatskii

1. A Single Exponential Binding Model

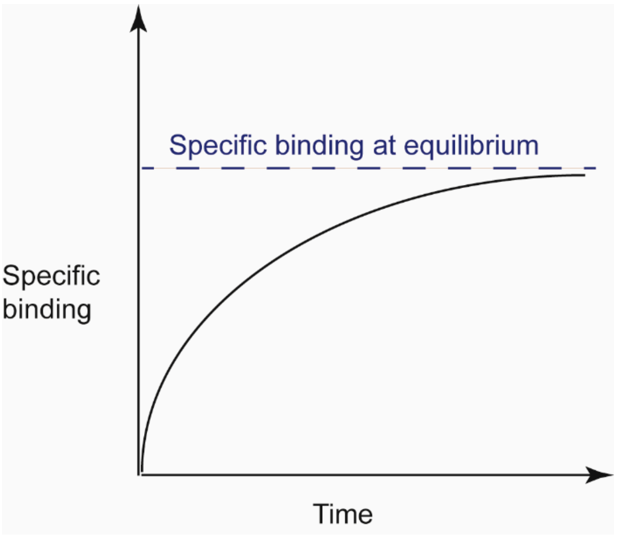

Figure S1. A single exponential binding model.

The simple binding model is derived from the law that the velocity of a chemical reaction is proportional to the concentrations of the ligand and receptor [1]. It is a simple approximation of a binding event assuming that all receptors are equally available for binding and it does not allow partial binding. This model also assumes that neither ligand not receptor is modified after binding implying reversibility of the reaction.

A reversible receptor–ligand interaction can be described by the following reaction:

$$\text{Receptor} + \text{Ligand} \leftrightarrow \text{Receptor} \cdot \text{Ligand}$$

Let Y be a concentration of receptor–ligand complexes at any time ($Y = [Ligand \cdot Receptor]$) and Y_{max} be a binding capacity of the receptor attached onto the surface. The rate of a complex formation (number of binding events per time) is proportional to the ligand concentration and amount of unoccupied receptor. Since not every ligand–receptor collision results in a complex formation, the association rate constant k_{on} has to be introduced:

$$[Receptor] \cdot [Ligand] \cdot k_{on} = (Y_{\max} - Y) \cdot [Ligand] \cdot k_{on}$$

The rate of a complex dissociation (number of dissociation events per time) is proportional to the complex concentration:

$$[Receptor \cdot Ligand] \cdot k_{off} = Y \cdot k_{off}$$

A dissociation constant K_D is defined as a ratio of dissociation and binding rate constants:

$$K_D = \frac{k_{off}}{k_{on}} = \frac{[Ligand] \cdot [Receptor]}{[Ligand \cdot Receptor]}$$

If now L is a thickness of the adsorbed layer and L_{max} is a maximum adlayer thickness proportional to the receptor concentration (when all sites are occupied), the net association rate

defined as a time derivative of the thickness *dL/dt* is equal to the difference between the forward binding and dissociation process:

$$dL/dt = (L_{\max} - L)\cdot[Ligand]\cdot k_{on} - L\cdot k_{off} = L_{\max}\cdot[Ligand]\cdot k_{on} - L\cdot([Ligand]\cdot k_{on} + k_{off})$$

After the integration, assuming that at t = 0 the adlayer thickness is zero, we obtain the expression for the simplest binding model described by a single exponential function:

$$L = \frac{L_{\max} k_{on}[Ligand]}{k_{on}[Ligand] + k_{off}}\cdot(1 - e^{-([Ligand]\cdot k_{on} + k_{off})t})$$

The adlayer thickness at the binding equilibrium obtained at infinite time is defined as follows:

$$L_{eq} = \frac{L_{\max} k_{on}[Ligand]}{k_{on}[Ligand] + k_{off}}$$

From this derivation the observed rate constant k_{obs} is an exponent in the exponential function and depends linearly on the ligand concentration. It is a measure of time needed to attain an equilibrium between association and dissociation processes during the complex formation. Thus the binding rate depends on association and dissociation rate constants, and linearly depends on the ligand concentration. A dissociation constant K_D can be calculated from the series of experiments with a variable concentration from the linear fit of k_{obs} as a ratio of k_{off}/k_{on}. If the simple binding model is not valid for a certain receptor–ligand system, the plot k_{obs} against the ligand concentration will deviate from a straight line.

If the binding does not obey a linear dependence we may infer that the binding is more complex than it can be described the simple model. In this case conformational changes, covalent or hydrogen bonds formation or other phenomena may take place.

2. Viability Justification for the Bacteria

For the experiment, bacteria were harvested fresh after an overnight incubation, dispersed in PBS, and pumped through the flow cell. Viability of the bacteria left in the suspension after the experiment was confirmed by growing new bacteria from the culture used in the experiment.

Viability of bacteria attached to the PC chip was proved using a standard two-color fluorescence assay (LIVE/DEAD® *Bac*Light Bacterial Viability Kit, Molecular Probes) based on different permeability into a bacterial membrane. A mixture of SYTO® 9 green-fluorescent nucleic acid stain and the red-fluorescent nucleic acid stain propidium iodide were used for the viability test. The SYTO 9 stain labels all bacteria that are incubated with it, whereas propidium iodide stains only bacteria with damaged membranes. After the kit application, living bacteria with intact membranes are stained in fluorescent green, whereas bacteria with damaged membranes appear red. The images show that only few bacteria are dead while almost all of them are alive. Since any freshly grown bacterial culture contains a small number of dead bacteria, the obtained results of fluorescence microscopy suggest that the technique used here preserves bacterial viability and does not result in bacterial death.

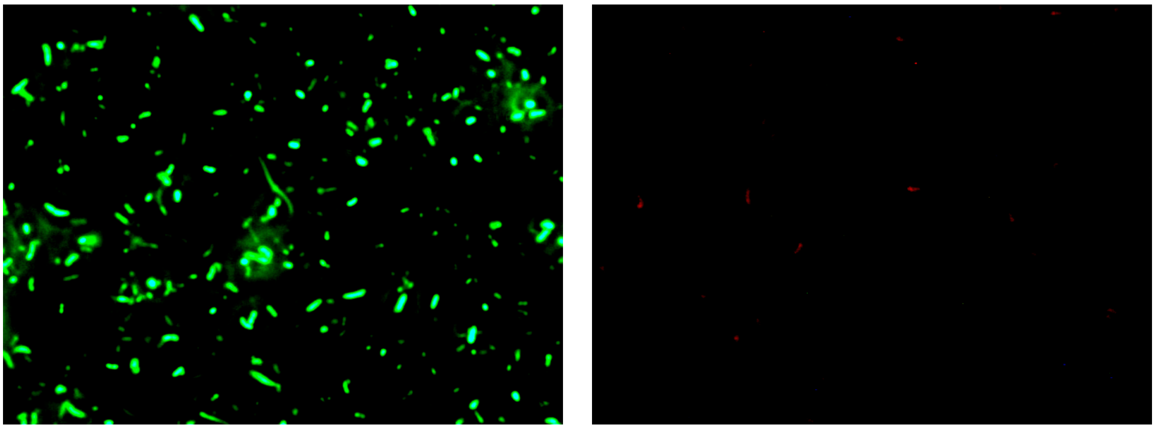

Figure S2. Verification of bacterial viability after the experiment: the chip was removed from the biosensor and incubated with the mixture of green and red fluorescent dyes. The left image shows all bacteria attached onto the PC chip stained by the green dye, the right one shows only dead bacteria among the total population.

3. Binding of Polyclonal Antibodies to Bacteria

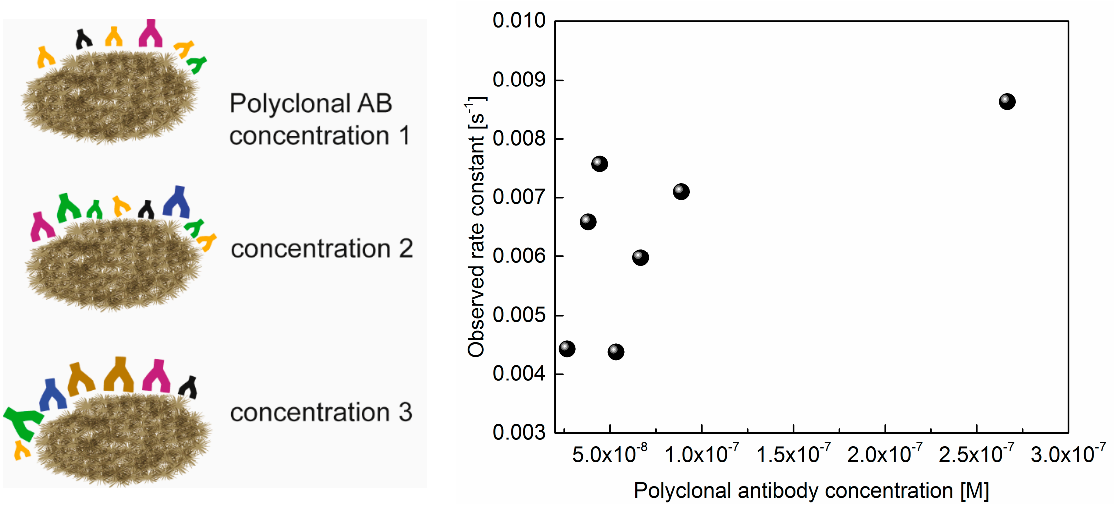

Figure S3. Left: Heterogeneity of antibody binding constants within an antibody population. Each subpopulation of antibodies in a polyclonal pool has different binding affinities (different colors) and antigen epitope specificities (an antibody size illustrates a binding constant value); **Right**: polyclonal antibody population contains various subpopulations with different binding constant and cannot be characterized by a single value.

A solution of polyclonal antibodies consists of several populations of heterogeneous antibodies with corresponding binding affinities and antigen epitope specificities within an antibody population. During an experiment with different antibody concentrations, a heterogeneous antibody sample is interacting with the bacteria as illustrated by different colors on Figure S3 (an antibody image size is associated with a different binding affinity). Due to the sample diversity, kinetic characteristics of each sample of different concentration are dependent on the presence of subpopulations of the antibodies in a polyclonal pool and, therefore, cannot be unified and described by a single binding constant.

References

1. Motulsky, H.; Christopoulos, A. *Fitting Models to Biological Data Using Linear and Nonlinear Regression*; Oxford University Press: Oxford, UK, 2004.
